# Ecosystem Barriers and Facilitators Linked to the Fear of Cancer Recurrence: An Umbrella Review

**DOI:** 10.3390/ijerph21081041

**Published:** 2024-08-07

**Authors:** Benjamin Caumeil, Nicolas Bazine, Axel Maugendre, Sarah Calvin

**Affiliations:** 1 Management Sport Cancer Laboratory (UR 20122035V), Faculty of Sport Sciences, Aix-Marseille University, 13008 Marseille, France; axel.maugendre@univ-amu.fr (A.M.); sarah.calvin@univ-amu.fr (S.C.); 2 Laboratoire 2LPN, UR7489, Université de Lorraine, 54000 Nancy, France

**Keywords:** cancer, fear of cancer recurrence, ecosystem, barriers, facilitators, oncology, umbrella review

## Abstract

The fear of cancer recurrence is an important topic in the healthcare field. In general, approximately 40% of survivors experience high levels of fear of recurrence. This study aims to fill this gap by synthesizing the findings of systematic reviews studies investigating ecosystems, correlates or predictors, and barriers and facilitators of fear of cancer recurrence among cancer survivors. An umbrella meta-synthesis was conducted using the following databases: MEDLINE, PsycINFO, PsycARTICLES, CINAHL, Business source premier, and SOCindex, ending in April 2024 with PRISMA methods. A total of 24 systematic reviews, representing 729 articles, were included in the study. In total, six ecosystems were identified, including family, work, friends, the healthcare system, caregivers, and religion. As part of this umbrella review, 55 specific ecosystemic factors were identified that may contribute to fear of cancer recurrence. Furthermore, the umbrella review identified 12 facilitators and 12 barriers related to fear of cancer recurrence. This umbrella meta-synthesis contributed significantly to our review’s strength in synthesizing the main ecosystem and its influence on fears of cancer recurrence. Understanding the interdependence of ecosystems should enable future research on intervention effectiveness or the development of interventions that could reduce the fear of cancer recurrence.

## 1. Introduction

Fear of recurrence (FCR) is a significant concern among cancer survivors [1]. Recent studies have shown that approximately 40% of survivors report high levels of fear of recurrence [2]. Specifically, FCR is linked to an increase in the likelihood of recurrence of cancer [3]. As a result, FCR is an important topic in public health. Our approach should be understood as a transdisciplinary one to make a contribution to an important public health issue. Several reviews have already been conducted to provide overall knowledge regarding the factors that contribute to a decrease in FCR [4]. Besides the classical antecedents (e.g., cancer types, demographics, and type of intervention), these reviews highlighted that the patient ecosystem is one of the most important factors contributing to FCR. Despite the lack of literature on the ecosystem, Broc et al. (2024) note that there has been an increased focus on the ecosystem in recent health research [5]. Notably, the identification can be via a comprehensive perspective of ecosystemic determinants, which refers to the variables affecting the health issues directly or indirectly [6]. A multitude of definitions and conceptualizations exist for the concept of ecosystem, also referred to as environment or context. Bronfenbrenner (2000) developed one of the most influential concepts of the environment in psychology [7]. The ecosystem was conceptualized as consisting of three subsystems: mesosystem, exosystem, and chronosystem. As the Mesosystem captures the context of the family, the exosystem captures the context beyond the family, and the last refers to the temporal context. Taking into account the conceptualization, the Dynamic Ecosystem Adaptation through Allostasis (DEA-A) model was utilized to capture the ecosystems in general health [6]. According to these authors, ecosystems can play a key role in how patients cope with illness and adjust to it. It may be possible to view these ecosystems as microsystems surrounded by macrosystems and exosystems, which are influenced by the chronosystem over time. A mesosystem enables the patient to be connected to these microsystems (e.g., work, family, healthcare, and religion). On top of this, the interrelationships between each microsystem play a significant role in determining the ontosystem of an individual. Thus, each system in the DEA-A model is affected by both its relationship to other systems and its own homeostasis and allostasis. Although this model is relevant to understanding the ecosystems of patients, it was not developed to take account of the FCR. Consequently, the purpose of this study was to integrate the Broc approach with another approach that had proven to be applicable in the context of FCR: the Maheu et al. (2019) [8] model, which was based on Lee-Jones’s work [9]. According to this model, there are two types of cues that influence fear of recurrence: internal and external cues. As opposed to external cues that represent the environment and external factors, which can increase worrying thoughts concerning a possible recurrence of the illness, internal cues involve somatic cues that reflect the threat of a return of the illness. As a result of the combination of the models of Broc et al. (2024) [6] and Maheu et al. (2019) [8], our objective was to identify the ecological determinants of the fear of recurrence. As mentioned previously, determinants refer to variables that can influence the fear of recurrence either indirectly or directly. Determinants can be divided into two types: barriers and facilitators. First, barriers capture the variables that increase fear of recurrence, for instance, researchers have shown that emotional distress increases fear of recurrence among cancer survivors [10]. Secondly, facilitators are variables that lower the fear of recurrence. Studies have shown that cancer survivors’ self-efficacy is associated with lower fears of recurrence [11]. Therefore, the purpose of this study is to determine the ecosystemic determinants of fear of recurrence through the identification of facilitators and barriers of fear of recurrence among cancer survivors. In light of this, we consider ecosystemic factors to be external cues that may contribute to an increase or decrease in FCR.

Despite previous individual reviews identifying external cues, ecosystemic factors, or external factors contributing to the fear of recurrence [12,13], a systematic review allowing for an overview of ecosystemic factors contributing to FCR remains lacking. It is important to note that most of these studies focused on specific interventions [14], components [15], or outcomes [16], which limits their ability to provide a comprehensive picture of the external factors leading to a decrease or an increase in FCR.

The purpose of this study was to fill this gap by synthesizing the findings of systematic review studies investigating any ecosystemic variables, correlates, or predictors of FCR among cancer survivors. As a second objective, the current study aimed to identify the facilitators and barriers that could be used to increase or reduce FCR among cancer survivors. In order to improve patient care and limit the adverse effects associated with FCR, it is essential to obtain some knowledge. Hence, we applied an umbrella review process that summarizes, assesses, and grades meta-analyses and systematic reviews providing a broader perspective on the findings regarding the influence of ecosystemic factors on FCR, thus evaluating the credibility of the relevant evidence. This research question was addressed using Page et al. (2021)’s Preferred Reporting Items for Systematic Reviews and Meta-Analyses (PRISMA) [17]. According to our knowledge, this meta-review represents one of the first attempts to synthesize empirical findings on ecosystemic factors leading to fear of recurrence. Thus, it could have a substantial contribution to the literature regarding fear of recurrence.

## 2. Methods

### 2.1. Study Design

In the present study, a systematic umbrella review was conducted. The umbrella method review is a new type of literature review that summarizes evidence from all systematic reviews and meta-analyses conducted on a broad topic [18]. The Joanna Briggs Institute (JBI) umbrella review method [19] provided an excellent framework for our umbrella meta-synthesis. There is a substantial amount of research on FCR and the ecosystem in cancer survivorship that supports the use of this methodology. Furthermore, the following key features of this review design were (i) gathering evidence from the multiple literature reviews or meta-analyses; (ii) including reviews based upon empirical studies rather than theoretical speculation or opinion; and (iii) summarizing existing reviews without resynthesizing the primary studies. It was reported according to the PRISMA guidelines [17].

### 2.2. Inclusion Criteria and Exclusion Criteria

Studies were included in case the following criteria were met: (1) the article must be written in either English or French; (2) the article must be a meta-analysis or systematic review–research studies (qualitative and quantitative); books, chapters, theses, editorials, guidelines, case studies, conference abstracts, indexes, and model proposals were excluded; and (3) studies should focus on FCR and their relationship with ecosystem in cancer survivorship.

Studies with the following characteristics were excluded from this review: (i) studies published in other languages than French or English, (ii) qualitative and quantitative studies, (iii) letters to editors, case studies, validation studies, or randomized control trials studies, and (iv) studies that did not emphasize ecological considerations and the fear of cancer recurrence.

### 2.3. Search Strategy

This search strategy was based on the SPIDER model [20]. In order to answer qualitative research questions, the SPIDER model consisted of five domains of interest, namely (1) Sample (S): patients with cancer or their caregivers; (2) Phenomena of Interest (PI): the fear of cancer recurrence; (3) Design (D): reviews that utilized qualitative, quantitative, or mixed methods designs; (4) Evaluation (E): N/A; and (5) Research (R): systematic reviews. As a result of the umbrella study’s nature, “evaluation” was not applied in the string. An umbrella meta-synthesis was conducted using the following databases: MEDLINE, PsycINFO, PsycARTICLES, CINAHL, Business Source Premier, and SOCindex, ending in April 2024 with PRISMA [17]. To enhance the sensitivity and inclusiveness of the search, the search terms included variations in MeSH terms and thesaurus keyword terms. As a first step, two authors (BC and NB) independently screened publications by titles and abstracts, while a third author (AM) independently double-checked the articles included. Following that, two authors (BC and NB) applied eligibility criteria to full-text articles in order to ensure consistency and reliability in the application of the criteria. The database search was performed by the first and last authors who screened for studies with the following keywords: (((“Relapse”) OR (“Recurrence”)) AND ((“Anxiety”) OR (“Concern”) OR (“Uncertainty”) OR (“Worry”) OR (“Apprehension”) OR (“Doubt”) OR (“Fear”) OR (“Dread”) OR (“Jitters”) OR (“Panic”) OR (“Scare”)) AND ((MM “Stakeholder*”) OR (“Doctor*”) OR (“Clinician*”) OR (MH “Physician*”) OR (MH “Hospital*”) OR (“Pair”) OR (“Family”) OR (“Nurs*”) OR (“Ecologic*”) OR (“Patient”) OR (“Team”) OR (“Work”) OR (“Friend*”) OR (“Context”) OR (“Health*”) OR (“Service”) OR (“System”) OR (“Supervisor*”) OR (“Colleague*”) OR (“Coworker*”) OR (“Organisation*”) OR (“Organization*”)) AND ((MM “Oncology”) OR (“Cancer”)) AND ((MM “Meta-analysis”) OR (“Meta-synthesis”) OR (MM “Systematic review”) OR (“Literature review”))). For the purpose of identifying articles within the databases, a Boolean formulation was employed. Furthermore, the gray literature (i.e., books) was taken into consideration. Finally, the bibliographies of each identified article were inspected, allowing us to extend the search as far as possible.

### 2.4. Data Extraction

Data extraction was performed in accordance with JBI guidelines [19]. Two authors (BC and NB) performed the extraction and a third author (AM) verified it for accuracy, in order to reduce the risk of bias. To ensure robustness, all of the articles were blindly reviewed by the first author and two other authors. The information extracted from each systematic review included the (1) authors; (2) year of the study; (3) country of each included studies; (4) objectives of the included review; (5) settings and context; (6) phenomena of interest; (7) number of database and sources searched; (8) data range of included studies; (9) number of studies, types of studies and country of origin of studies included in each review; (10) appraisal instrument and rating; (11) outcomes of interest reported relevant to the umbrella review question; (12) key synthesis finding methods employed to synthesize the evidence; and (13) comments/notes the umbrella review authors may have regarding any included study. The results of the systematic data extraction from the included studies are summarized in Table 1. We gathered data describing the following: authors (year of publication), country of included studies, number of included studies with their respective population and design, determinants, ecosystems, barriers, facilitators, and methodological quality.

### 2.5. Assessment of Methodological Quality

The papers included in the final analysis were blindly assessed by two of the authors (BC and NB) using the Joanna Briggs Institute Critical Appraisal Checklist for Systematic Reviews and Research Synthesis. When there is disagreement about the quality of an article, a third author’s opinion (SC) is sought in order to reach a final decision.

The Joanna Briggs Institute assessment tool consists of 11 questions. The items are evaluated as Yes, No, Unclear, or Not applicable. One point is awarded for the answer Yes, while zero points are awarded for all other answers. Based on the sum of points, the papers were classified into three categories: low quality (0–4), moderate quality (5–8), and high quality (9–11) [42,43]. Prior to the review, all authors agreed on a minimum quality threshold to preserve medium- and high-quality articles. Consequently, the literature reviews that were considered low quality were excluded from the analysis to preserve articles possessing high methodological qualities. The results are summarized in Table 2.

### 2.6. Data Synthesis

First, the data from the included articles will be presented, along with an analysis of the methodological qualities of these studies. Second, the characteristics, samples, and methods of the studies will be presented. Based on the main purpose of our review, we have organized our arguments as follows: (i) types of ecosystems, (ii) determinants of ecosystems, (iii) facilitators, and (iv) barriers. The information has been synthesized by the ecosystem and classified in terms of the number of studies that have been conducted on it (determinants, facilitators, and barriers).

## 3. Results

### 3.1. Study Inclusion

**Figure 1:**
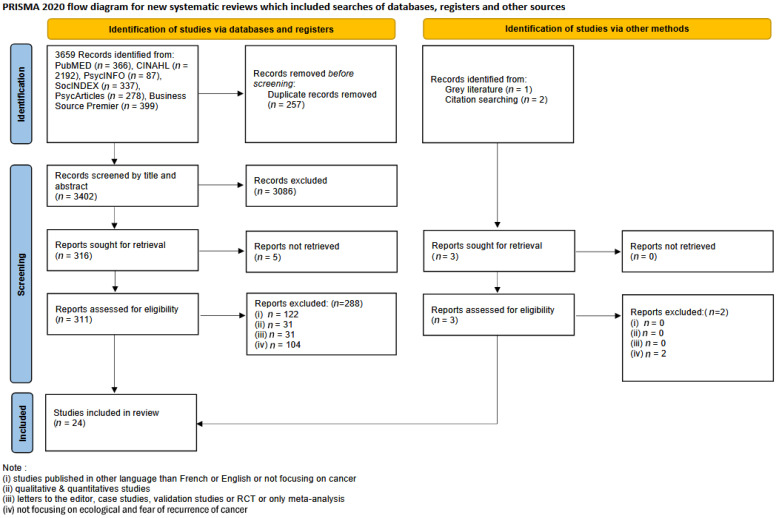
PRISMA 2020 flow diagram for new systematic reviews that included searches of databases, registers, and other sources.

A total of 3659 articles were identified, PubMED (*n* = 366), CINAHL (*n* = 2192), PsycINFO (*n* = 87), SocINDEX (*n* = 337), PsycArticles (*n* = 278), and Business Source Premier (*n* = 399). A further three records were identified from the grey literature. Prior to the first stage, 257 duplicates were removed. A total of 3402 articles were screened for the first stage based on titles and abstracts. A total of 3086 records were excluded from the analysis because they did not meet the inclusion criteria and five records were not retrieved. In total, 311 articles were assessed for eligibility for the second stage. Among the 311 articles, 24 met the inclusion criteria. In the end, these 24 articles were selected as the final set of articles for analysis. A PRISMA flow diagram is presented in Figure 1. For specific database details, see Table 3.

### 3.2. Characteristics of Included Studies

**Figure 2:**
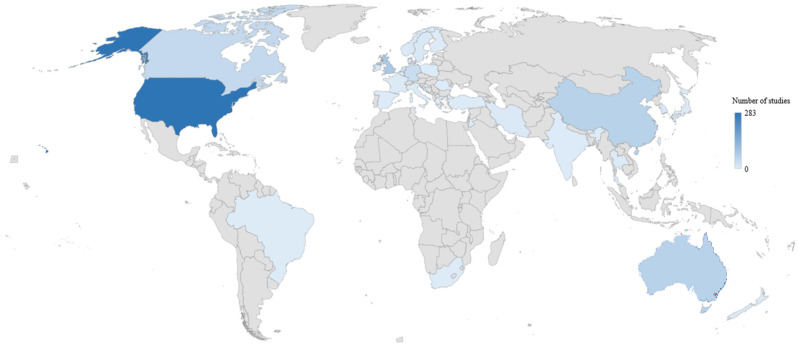
Distribution of the country of origin across the included studies.

In total, 24 systematic reviews represented 729 articles (see Table 1). According to these 729 articles, the top 5 countries were the United States (*n* = 283), the United Kingdom (*n* = 82), China (*n* = 63), Australia (*n* = 61), and Canada (*n* = 42). Figure 2 illustrates the range of countries in which the studies were conducted. In addition, the top 5 types of studies included were cross-sectional (*n* = 302), qualitative (*n* = 111), quantitative (*n* = 85), longitudinal (*n* = 77), and intervention (*n* = 25). Participants in all of the systematic reviews represented a total of 195,531 participants, divided into patients and caregivers.

### 3.3. Methodological Quality

According to the Joanna Briggs Institute assessment tool, 12 of the 24 studies included in this Umbrella Review were rated as high quality, 10 as moderate quality, and 2 as low quality. Accordingly, based on the quality assessment criteria, the two articles with low quality were excluded from the analyses [27,33]. Detailed information regarding the critical assessment and risk of bias for the included studies is provided in Table 2.

### 3.4. Findings of the Review

#### 3.4.1. Ecosystem

A total of six distinct ecosystems were identified, including family (e.g., children, siblings, or partners), work (colleagues AND superiors), friends, the health system and health providers (such as psychologists and social assistants), caregivers (such as physicians and nurses), and religion (such as churches and communities).

With regard to the ecosystem, 13 research studies [22,23,25,26,28,30,31,34,35,36,37,38,39] were conducted on family and the interaction between family and the patient and its effects on FCR. Most of these studies showed both positive and negative effects of FCR. For example, those with marital insecurities [22] had a higher FCR than those with a supportive and understanding partner [39]. Furthermore, four studies [2,32,37,38] examined the effects of the work context on FCR. It has been suggested that employment has some protective effects [38], as well as increasing the FCR scores [2]. Furthermore, 7 studies [22,25,28,29,30,31,36] examined the effect of peers and friends on FCR. For instance, social functioning can have protective effects [38] but it can also lead to an increase in FCR scores [2]. Additionally, six studies examined the role of the health system and care support [3,21,25,28,38,41]. The lack of understanding of the healthcare system, for example, may be a significant predictor of the FCR score [21], whereas the provision of continuity of care may be a protective factor for the FCR score [28]. Furthermore, 13 studies [3,14,24,25,26,28,29,31,35,37,38,39,40] have highlighted the role of caregivers. One ambivalent example was communication. Thus, when the caregiver took the time to explain the care, it could be protective of FCR [35]. It is also at risk of being predictive of FCR if it is performed without taking into account the patient’s young age or low education level [3]. Lastly, five studies [2,22,37,38,41] have demonstrated the importance of religion on FCR. For example, religious coping has been shown to lower FCR scores [37] but struggling spirituality has also been shown to have a significant effect on improved FCR scores [2].

#### 3.4.2. Determinants

This umbrella review highlighted a set of 55 specific ecosystemic antecedents of FCR. Using the Lee-Jones (1997) model [9], which has been expanded by Maheu et al. (2019) [8], we defined two categories of cues: internal cues and external cues. There are several factors that can be attributed to internal cues, such as self-esteem and self-efficacy, which are the most commonly studied in systematic reviews [22,38] of uncertainty [28,34] and emotional distress [2,35,38]. In contrast, for external cues, we can identify six sets of factors found within the ecosystem. These factors include family, work context, religion, friends, the health system and care support, and caregivers.

Firstly, this review identified that the presence of a partner is an important determinant of FCR [3,22,28,35,38]. More precisely, our review has identified that factors such as not having a partner [23,25,38] or sexuality problems are related to higher FCR levels [22,28,31,37,38]. Furthermore, FCR has been linked to family [26,31,38] or the presence of children [23,25,38].

Secondly, our review indicates that the quality of relationships with friends is associated with a reduction in FCR [2,38]. Furthermore, peers who have undergone similar illnesses exhibit a lower FCR [22,29].

Regarding the work context, two major determinants were identified. In addition to being employed [2,23,31,38], financial concerns are positively associated with FCR [28,37].

Fourth, our review has demonstrated that healthcare support and the health system play a significant role in FCR. In six studies [25,28,34,37,38,41], health system’s accessibility was found to be a significant determinant of FCR. We have identified three critical determinants of FCR as the caregiver [14,35,40], the annual checkup or appointments with a health professional [14,23,26,38,39,41], and, finally, the communication with the caregivers [3,24,31].

Lastly, our review identified religion as an important ecosystemic factor influencing FCR. According to three studies [2,37,38], spirituality and church community are important predictors of FCR.

Finally, six transversal ecosystemic factors were identified through our umbrella. First, isolation was identified as a factor in two studies [22,31]. The second factor identified was social support which was studied in 12 studies [2,3,22,25,30,31,32,34,35,36,38,39]. Third, coping skills were examined in six studies [23,24,35,36,37,41]. The last factor identified was the information provided to the patients, which was examined in nine studies [21,22,23,28,29,31,34,36,37] (See Table 1).

### 3.5. Barriers and Facilitators

The ecosystemic determinants were divided into two categories: barriers and facilitators. According to some authors, barriers and facilitators may be influenced by the same factor [44].

In terms of barriers, this umbrella review identified 12 different factors. First, three barriers were identified in the family ecosystem. These include dysfunctional family [35,38], sexuality [36,38], and the difficulty of initiating conversations about sexuality [31,37]. According to some authors, these barriers can be attributed to the family sphere [36,37,38], while others place them in connection with health professionals (e.g., healthcare professionals, and psychologists) [31]. Second, one factor was associated with the friendship ecosystem, namely social constraints [25,34,35]. Additionally, our review identified two additional barriers related to the work context ecosystem. The first barrier is financial constraints [22,41], whereas the second is employment status: to be employed or not [2,32,38]. There are two additional barriers related to the health system ecosystem and caregivers ecosystem, such as the lack of psychological support [2,34] and the lack of information from caregivers [3,29,36,37]. There were two barriers identified regarding the intrapersonal level, which are dysfunctional coping strategies [24,41] and distress [35,38,40]. A final common barrier in the family, friendly, and intrapersonal ecosystems was identified as loneliness or isolation [35,37].

As for facilitators, there are 12 major facilitators that are frequently cited in systematic reviews. In the family sphere, one facilitator is the quality of family interaction [36,37,39]. The health system and supportive care have two facilitators for reducing FCR, namely interventions (e.g., cognitive behavioral therapy, group therapy, and counseling) [2,23,24,31,38,41], as well as support needs/support groups [2,25,31]. Another mechanism for reducing the FCR is the ecosystem of religion with religious/spiritual practices [2,38]. There is also a facilitator for the medical field and caregivers with medical follow-up [35,37,39]. A total of five facilitators were identified as intrapersonal factors including self-motivation [3,22], self-efficacy [24,25,38], problem-focused coping strategies [24,35,37,38], communication [3,24,28,35], and patient-reported outcomes (e.g., distress, anxiety, depression, and quality of life) [28,31,35,38]. The remaining two facilitators are common to the work, friendly, family spheres, namely support from family or community [21,31,37], and social support [30,38,39].

## 4. Discussion

Ecosystems have important implications on the fear of recurrence. This meta-review was conducted in order to identify ecosystemic factors contributing to a decrease or an increase in FCR. In order to provide a comprehensive assessment of ecosystemic factors contributing to FCR, we sought to review the existing literature and conduct a systematic review.

This review was conducted through an umbrella metareview analysis. While this type of method is still relatively new, it allows the synthesis of information from systematic reviews and meta-analyses while retaining the most significant information [18]. Consequently, six main ecosystem families were identified, as well as specific factors that determine the FCR within each ecosystem family.

### 4.1. Ecosystem

The strength of our review was to synthesize the main ecosystem through this umbrella meta-synthesis to influence FCR. As a result of our review, six different ecosystems have been identified: family, work, friends and peers, the health system and health providers, caregivers, and religion. An additional system was identified that is rather personal to the individual, which will be referred to as the intrapersonal system. The majority of studies regarding FCR were conducted on family ecosystems and caregivers. A number of studies have shown that children, partners, and family caregivers tend to reduce FCR [22,25,26,37]. In addition, it is important to note that cancer patients have a low representation of the work ecosystem due to their advanced age [45]. In light of this, very few studies have been conducted on younger populations, including adolescents and young adults [46], despite the importance of returning to work for these individuals [47] or of socializing with peers at school [48].

On the basis of the models developed [6,8], we have identified a number of ecosystemic factors that influence FCR.

### 4.2. Barriers and Facilitators

In addition, our umbrella review has identified many barriers and facilitators to the previous ecosystems. Barriers tend to be more prevalent in certain ecosystems, such as family and work environments, but also within the individual. These family barriers may be more prominent because the patient’s relationship with his family (e.g., relationship quality with their husbands and children, sexuality, and presence of a partner) also poses a significant risk of psychological distress for the family [49,50] as well as a resource to cope with the threat of the disease returning. Indeed, psychological distress may affect the couple’s relationship within the family, increasing the caregiver’s burden. Thus, the caregiver could see themselves as increasingly vulnerable to a situation they are not experiencing (i.e., having cancer) and this may result in a physical or psychological collapse [51], which could lead to the appearance of real psychological distress for the family. It is therefore possible to prevent psychological distress for a spouse and partner when targeted interventions are implemented in the dyadic husband–wife relationship [52]. Barriers to work are generally represented by insecurities pertaining to financial matters or by the loss of a job, both of which increase the risk of FCR. In this manner, studies have identified specific factors influencing the decision to return to work and remain in employment as well as identifying obstacles to such a return [53]. It should also be noted that the employed factor is ambivalent; it is both predictive of an increased FCR score [2] as well as protective against it [38]. In that case, it may be possible to find an explanation that is more closely related to the work environment. In fact, if the patient perceives their colleagues as being benevolent or supportive, they might consider returning to work more serenely rather than in a heavy work atmosphere that would slow them down. Therefore, we can consider, in both cases, a reduction in FCR since the patient is adequately satisfied with their needs and perceptions of the situation, unlike situations where the patient is forced to return to work due to financial concerns, which could be problematic in a challenging environment [28]. The potential obstacles to reducing FCR include dysfunctional coping strategies and significant psychological distress, which constitute the conditions conducive to the manifestation of high levels of FCR, as several existing psychological models indicate [8,54]. Furthermore, there are facilitators available today to reduce FCR levels, thereby promoting homeostasis. It is possible to identify three major categories of ecosystems, namely those involving supportive care, those relating to intrapersonal factors, and those that are common to several ecosystems. As a result, supportive care, such as interventions and counseling, is most likely to result in a reduction in FCR. A number of meta-analyses that focus specifically on FCR interventions have found this result [55,56]. Despite the fact that certain interventions appear to reduce FCR more directly than others, some interventions act as mediators and/or moderators of the relationship by promoting coping strategies focused on the problem and thus indirectly reducing FCR in a second instance [57]. Consequently, intrapersonal factors such as problem-focused coping strategies, self-efficacy, patient-reported outcomes, motivation, and communication contribute to the reduction in FCR. As a result, interventions should be targeted at one or more components of FCR in order to reduce it. In recent years, there have been an increasing number of interventions aimed at reducing the FCR [58]. However, each intervention does not affect the same cognitive and somatic processes in the same way. In fact, some approaches, such as mindfulness, focus more on the somatic consequences, while cognitive behavioral therapy tends to focus on cognitions [59]. Finally, social support and support groups have been identified as important elements in reducing FCR in several ecosystems [60,61]. Indeed, social support is one of the most cited determinants in studies aimed at reducing FCR [62,63]. From the perspective of all barriers and facilitators identified in the literature, we currently have too few effective interventions. Considering recent studies, it is imperative that we accelerate the development of effective interventions based on the barriers and facilitators identified in the ecosystems in order to facilitate the patient’s ability to find the homeostasis that is most conducive to their well-being [5].

### 4.3. Determinants

Several determinants have been found to predict or be linked to the FCR. The presence of the partner or not, as well as sexuality, the quality of relationships with friends, or peers who have experienced similar illnesses, employment, financial concerns, access to health systems, caregivers, annual check-ups with a health professional, communication with caregivers, spirituality and church community, isolation, social support, coping, and information are all factors related to FCR. Several of these determinants were extensively described in studies [13,64], some of which are more difficult to alter than others. However, recommendations are emerging concerning the need to directly or indirectly act upon these determinants in relation to barriers and facilitators. In some cases, they may lead to a spiral of negative events that negatively impact the patient and may make it more difficult for them to cope alone [65]. Consequently, researchers suggest that increased efforts should be made for prevention [66,67] and for changes in patient behavior in order to overcome the negative consequences and avoid FCR [68].

### 4.4. Characteristics

According to the characteristics of the included studies, most of them were conducted in developed countries. Despite this, major differences exist between health systems [69]. Based on our findings, most studies have been conducted in developed countries, with a clear dominance of publications from the United States, United Kingdom, and Australian countries [70]. By 2040, the Global Cancer Observatory predicts a 47% increase in cancer incidence, reaching approximately 28.4 million cases [71]. Consequently, transitional or low Human Development Index countries will experience the largest increase and the greatest burden of the disease. However, their cancer care and control infrastructures are among the least developed despite the importance of psychosocial and medical support [72]. There is a possibility that this may explain the differences in focus between studies conducted in the United States that provide more attention to the people around the patient and those conducted in France, which feature a healthcare system that focuses on the patient and those around them [73]. It is also noteworthy that most of the included studies are quantitative research studies involving longitudinal or cross-sectional design. In only one-seventh of the studies included, qualitative data were sought. There is no doubt that both types of studies are important and provide a variety of perspectives [74]. In addition, a wide variety of cancers were included in the studies and the management may vary depending on a cancer’s grade, location, or type. However, all of them can cause higher FCR levels [1]. Accordingly, a probable better understanding based on the health system as well as the study method used to collect, exploit, and analyze data, based on the type and grade of cancer, could enable better refocusing of the patient within their ecosystem and enable more individualized interventions to reduce FCR.

### 4.5. Limits

A major limitation of this study is the choice to group ecosystems and classify factors, barriers, and facilitators within them. As a matter of fact, this represents a significant bias since it requires agreement on the boundaries between one ecosystem and another. In fact, other existing models engage in a more ecological approach to the direct relationship with the patient [6]. Another limitation is the number of studies that have been included. Several systematic reviews have cited certain studies and these studies have been counted more than once. Furthermore, little research has been conducted to address the specific needs of AYA audiences, which present interest in the work ecosystem, suggesting an important opportunity for improving research to meet the needs of such a population. It is important to consider an additional limitation when assessing the quality of systematic reviews and research syntheses using the Joanna Briggs Institute checklist, which can be improved by incorporating intermediate yes or no boxes into the assessment process. Another important limitation is the date range of the included studies. The period covered by our study ranges from 2008 to 2024. This choice can be considered as a limitation because it is arbitrary rather than motivated. When conducting systematic reviews, limiting the date range is crucial because it prevents outdated information from being included in the review. It is the nature of healthcare to continually refine interventions, guidelines, and treatments. Excluding older studies through a well-defined date range restriction becomes a deliberate choice that aims to eliminate information that may no longer be relevant or reflective of current best practices. Careful curation of systematic reviews is essential to ensure their relevance and their usefulness in contemporary healthcare settings. Lastly, the exclusion of only meta-analysis studies may have hindered the identification of additional determinants, levers, or obstacles to FCR.

### 4.6. Clinical Implications and Perspectives

Based on the results of the umbrella review, six important ecosystems could be useful in understanding the patient holistically. To prevent the onset of FCR and/or manage its impact, healthcare professionals should systematically question patients as soon as the illness is diagnosed. This approach aims to clearly identify not only the patient’s support needs but also the state (extent of their network, varieties, etc.) and functioning of their ecosystem and satisfaction with it. The development of support tools aimed at diagnosing the ecosystem may improve referrals to support systems (both formal and informal) that can help patients manage their FCR. Oncologists and paramedical staff often face significant challenges in addressing cancer patients’ relational and social issues and guiding them toward the most suitable solutions. The main difficulty lies in the fact that many caregivers do not feel competent or effective in understanding the determinants of fear of recurrence or in assessing its intensity [75]. Although standardized and validated instruments, such as the Relationship Quality Inventory [76], the Social Network Index [77], and the Social Support Behavior Inventory [78], can measure the quality of patients’ relationships with family or caregivers, caregivers’ perceived lack of competence limits their ability to provide adequate support and effectively identify the most beneficial interventions or networks for patients. The development of a single PROM (Patient-Reported Outcome Measures) tool adapted to clinical practice would provide a comprehensive assessment approach. This tool would evaluate the support needed to cope with FCR, the patient’s preferences regarding the type of support, the availability of resources, and the barriers to their mobilization. For researchers, systematic evaluations could be used to reflect on, implement, and personalize effective psychological and social interventions in the real-life environment of patients, as is strongly recommended today [67,79]. Consequently, this approach could be of great importance in preventing the psychosocial impact of FCR on patients’ quality of life and survival.

## 5. Conclusions

This study emphasizes the importance of taking into consideration the patient’s environmental factors when evaluating the appearance of fear of cancer recurrence. The identification of barriers and facilitators has enabled the development of patient care by recommending adapted but, above all, individualized interventions in order to reduce the fear of cancer recurrence. Understanding the interdependence of ecosystems should enable future research on intervention effectiveness or the development of new interventions that could reduce the fear of cancer recurrence. Therefore, future research should focus on determining which are the most effective interventions for reducing the fear of cancer recurrence by proposing rigorous methodologies such as randomized controlled trials in order to minimize the potential for bias associated with other types of interventional studies. Furthermore, studies that combine qualitative and quantitative approaches to better understand the dynamics of the ecosystem around the patient may be able to provide a better understanding of the evolution of FCR over time.

## Figures and Tables

**Table 1:** Overview and description of the included systematic reviews.

Authors (Year)	Country of Included Studies	Number of Studies Included/Number of Participants/Design	Determinants	Ecosystems	Barriers	Facilitators	Quality Assessment
Anderson et al. (2021) [21]	USA (16 studies) Australia (1 study) Canada (1 study) UK (1 study)	19 included articles *N* = 16,672 patients Quantitatives (*n* = 14) Qualitatives (*n* = 4) Mixed methods (*n* = 1)	Meaning External factors Poorer understanding of the healthcare system	External factors Healthcare system	Poorer understanding of the healthcare system	Cultural beliefs Support from family and community	Moderate
Bamidele et al. (2022) [22]	UK (5 studies) USA (4 Studies) Canada (1 study)	10 included articles *N* = 139 Black men with different cancer Qualitatives (*n* = 10)	Isolation from social contacts injured self-esteem marital insecurities desired more information from their healthcare providers to manage these challenges partner (support) family wider social networks (peers who undergone similar illness experience)	Unstructured: partner peers family Structured church community community cultural group online support group	Self as a barrier underpinned by masculinity concerns and personality types Cultural stigmatisation of masculine sexual dysfunction Healthcare system, structure and process as Barriers Financial and physical health challenge	Influence of others Self-motivation as informed by the illness experience and treatment side-effects	High
Crist and Grunfeld (2013) [23]	USA (17 studies) UK (8 studies) Netherlands (5 studies) Canada (4 studies) Germany (3 studies) Australia (2 studies) Japan (1 study) Norway (1 study) South Africa (1 study) South Korea (1 study)	43 included articles *N* = 13,953 patients with different cancer Cross-sectional (*n* = 16) Longitudinal (*n* = 11) RCT (*n* = 6) Prospective (*n* = 6) Case control (*n* = 1) Prospective + Cross-sectional (*n* = 1) Retrospective (*n* = 1) Secondary analysis (n = 1)	Coping Medical information Annual check-up Concurrent family stressors Unpartnered patients Having children Employed Marital status, family ressources are unrelated to FCR	Family and psychological factors	Having young children	Interventions targeting illness perceptions and inappropriate checking behaviour using a form of cognitive behavioural therapy	Moderate
Dawson et al. (2016) [24]	USA (4 studies) Germany (1 study) Canada (1 study) France (1 study)	7 included articles *N* = 719 participants RCT (*n* = 3) RCT pilot feasability (*n* = 1) Longitudinal (*n* = 1) Qualitative (*n* = 1) Case study (*n* = 1)	Coping Communication Emotional support	Oncology Physicians/providers	Dysfonctional coping strategies	Awareness of available resources perceived helpfulness of the resources received better coping and problem solving Communication Counseling Group Therapy Self-efficacy	Moderate
Deckx et al. (2021) [14]	UK (10 studies) USA (9 studies) Canada (5 studies) Netherlands (4 studies) Australia (4 studies) Denmark (1 study)	33 included articles *N* = 39,395 patients Observational quantitative (*n* = 19) Intervention (*n* = 9) Qualitative (*n* = 5)	Number of visits of general practitioners	General practitioners		General practionners as gatekeeping	High
Gormley et al. (2022) [25]	USA (4 studies) Australia (4 studies) Canada (3 studies) UK (2 studies)	13 included articles *N* = 2742 Participants Cross-sectionnal (*n* = 10) Qualitative (*n* = 2) Longitudinal (*n* = 1)	No association with religion No association with Previous cancer experience in friend/relative, recurrence in friend or relative Motherhood status Social support Cognitive behavioral factors (e.g., cognitive processing, metacognition, illness intrusiveness) Surveillance: Health Behaviours (e.g., adherence to breast self-examination and mammography schedule, unscheduled visits to healthcare provider) Parenting stress	Family Children Friends Healthcare provider (General Practitionner) Healthcare system	Breast self-examinations obsessively more frequent unscheduled healthcare provider visits Avoidance of surveillance visits Social constraints Metacognitions (believing that worry is harmful, seeking control over cognition)	Mammography or Ultrasound in the past 12 months Support needs/Support groups Confidence in disease management Self-efficacy	High
Hampton et al. (2024) [26]	South Korea (5 studies) China (5 studies) USA (4 studies) Canada (3 studies) Sweden (3 studies) Australia (2 studies) Many countries (2 studies) UK (1 study) France (1 study) Romania (1 study) India (1 study) Germany (1 study) Croatia (1 study)	31 included articles *N* = 11,857 patients Cross-sectional (*n* = 25) Mixed-Methods (*n* = 5) 1 RCT (*n* = 1)	Higher level of education Parenthood Upcoming appointment related to cancer Personal experience with cancer related death	Healthcare Parenthood	Distrust in healthcare Upcoming appointment related to cancer Personal experience with cancer related death	Higher level of education Planing a daily routine	High
Koch et al. (2013) [3]	USA (14 studies) Germany (1 study) Netherlands (1 study) Norway (1 study)	17 included articles *N* = 6673 patients with different cancer Longitudinal (*n* = 11) Cross-sectionnal (*n* = 4) Qualitative (*n* = 2)	Partnership Never fathered children Social Support Family distress Patient provider communication	Clinicians Care providers	Communicating more with providers raised FCR	Survivors care plans implemented in the US and UK Remaining fear should be used as a motivation for self-care Patient provider communiction should be targeted to the needs of the individual patients (e.g., younger age, less education)	Moderate
Lavery and O’Hea (2010) [27]	USA (6 studies) UK (1 study) India (1 study) Brazil (1 study) Germany (1 study)	10 included articles *N* = 904 participants (2 studies did NR) Qualitative (*n* = 4) Longitudinal (*n* = 3) Cross-sectionnal (*n* = 2) Pilot study (*n* = 1)		Public religious practices	NR (not reported)	Public religious practices Private religious coping	Low
Lisy et al. (2019) [28]	Australia (17 studies)	17 included articles *N* = 5925 patients with different cancer Cross-sectionnal (*n* = 15) Longitudinal (*n* = 2)	Information needs and medical care issues, emotional and relationship issues Physical and daily living, sexuality, patient care and support, health system, information Access and continuity of care, relationships, financial concerns, information Structure of care, process of care, relationships, information, daily living, school/occupational Information/support, sexual, future needs Cope with the uncertainty about the future Worries and emotions of parteners, family members, friends Support partners and family members Unmeet needs for help with changes to sexual feelings and relationships	Family (Partners and family members) Friends Supportive care Healthcare professionals Peer support	Unmeet needs within the supportive care domain were access and information about complementary and alternative therapies Healthcare professionals communicated to coordinate care Empoyed Less social support	Patient-reported outcomes (improved communication between patients and their treating teams, increased identification and management of symptoms, increased health-related quality of life, increased patient satisfaction, and reduced emergency departement utilisation)	Moderate
Liu et al. (2019) [29]	USA (6 studies) UK (5 studies) Australia (2 studies) Germany (1 study) Netherlands (1 study) Ireland (1 study)	16 included articles *N* = 3885 patients Intervention (*n* = 16)	Information needs/provision Unsatisfaction with information provided Patient-centred assessment of fears Provision of risk information by the oncologist, reassurance/normalisation, referral to cancer support groups and online resources, peer counselling and psychologist	Peer counselling Oncologist Psychologist referral	Unsatisfaction with information provided	Fill information needs/provision	Moderate
Lu et al. (2023) [30]	China (37 studies)	37 included articles *N* = 8190 patients Cross-sectionnal (*n* = 36) Secondary analysis (*n* = 1)	Social support	Family Friend Other	NR (not reported)	Social support	High
Luo et al. (2024) [31]	China (11 studies) UK (9 studies) Australia (8 studies) US (8 studies) Netherlands (4 studies) Sweden (2 studies) Canada (2 studies) Italy (1 study) New Zealand (1 study) Norway (1 study) Singapore (1 study) Spain (1 study) South Korea (1 study)	50 included articles *N* = 6566 colorectal cancer patients Quantitative (*n* = 33) Qualitative (*n* = 15) Mix-method (*n* = 2)	Information needs of family, and support for family members, family relationships, family worries, fertility concerns, and concerns for family future. Need for help with experience of social isolation, inefficient social support, and concerns of social relationship. Problems with sexuality, sexual dysfunction and sexual relationships. Employment issues Need for help to provide information about CRC (e.g., diagnosis, treatment, follow-up, knowledge of self-care and health promotion), reliable and various information, resource, and support with preferences in information (e.g., updated, understandable or personalized information). Need for help with better coordination among healthcare professionals, better communication between patients and clinicians, and satisfaction with care	Family-related Social or relationship Caregiver/Practicionner Healthcare professionnal	survivors and their spouses feel embarrassed to discuss sexuality	Patient-reported outcomes (distress, anxiety, depression, and quality of life) Family support Sexual healthcare (healthcare professionals, companions and patients) Peer Support group Interventions	High
McGeechan et al. (2022) [32]	US (16 studies) UK (10 studies) Canada (4 studies) Australia (4 studies) Netherlands (3 studies) Sweden (2 studies) Iran (2 studies) Norway (1 study) Singapore (1 study) China (1 study) Hong Kong (1 study) Ireland (1 study) Multi-countries (1 study)	47 included articles *N* = 786 patients colorectal cancer Qualitatives (*n* = 46) Secondary analysis (*n* = 1)	Social support	Work	Return ton work can be challenging (physical limitations, loss of stamina, anxiety)	Supportive colleagues and employers Recognition that physical limitations may necessitate a revaluation of what is feasibly possible for patients to carry out	Moderate
Mistchke (2008) [33]	NR	48 articles included NR Patients Research studies (*n* = 37) Theoretical articles (*n* = 11)	Experience of couples Family Family members’ fear Social support	Family interaction	Dysfunctional family Loss of mother daughter relationship	Quality of family interaction	Low
Neves et al. (2023) [34]	US (11 studies) Ireland (1 study) China (1 study) Belgium (1 study) Australia (1 study) Canada (1 study)	16 included articles N = 1896 caregivers Cross-sectionnal (*n* = 10) Qualitatives (*n* = 4) Longitudinal (*n* = 2)	Hypervigilant Lack of acces to services or supports Readjusting socially Social constraints Intolerance of uncertainty Distress (anxiety, suicidal thought, trying to suppress how they felt)	Family (parents of AYA) Married mother	Lack of psychological support Quality of parent-child-relationship Social constraints (i.e., more difficulty disclosing negative thoughts, concerns, and feelings to others)	Watching out for physical and emotional changes and symptoms, seeking medical attention when symptoms appeared, asking how their child was feeling) Information Making lifestyle changes Talking about their concerns Praying Taking it day by day	Moderate
O’Rourke et al. (2021) [35]	USA (12 studies) UK (3 studies) Ireland (1 study) Netherlands (1 study) China (1 study) Taiwan (1 study)	19 included articles *N* = 2887 patients with different cancer Cross-sectional (*n* = 11) Longitudinal (*n* = 8)	Partners Caregivers Ethnicity Emotional distress Interpersonal factors Social support and conselling (coping strategies)	Partners Caregivers Family members Interpersonal factors	Distress Unsupportive partners Social constraints Loneliness Relationship quality Spouse negative affect	Quality of life Coping strategies Medical appointments Relationship quality Communication	High
Schmid-Büchi et al. (2008) [36]	US (9 studies) Australia (7 studies) UK (3 studies) Canada (1 study)	20 included articles *N* = 3014 patients with different cancer Cross-sectional (*n* = 12) Qualitatives (*n* = 3) Quantitatives (*n* = 3) Longitudinal (*n* = 1) Focus group (*n* = 1)	Coping (informal coping style: seeking or not seeking information) Information Post-traumatic growth Social support Felt down	Partners Friends	Women with newly-diagnosed breast cancer and adjuvant therapy experienced greater role limitations and impairment in their social functioning than women with stable disease Sexuality Overcoming barriers in obtaining health information	Partners have a mutual influence on one another Relationship improved	Moderate
Schubach et al. (2024) [37]	USA (7 studies) UK (3 studies) Netherlands (2 studies) Poland (2 studies) China (2 studies) Greece (1 study) South Korea (1 study) Japan (1 study) Canada (1 study) Ireland (1 study)	21 included articles *N* = 3654 participants Qualitative studies (*n* = 2) Cross-sectional (*n* = 16) Mixed methods (*n* = 3)	Information Sexual well-being Needing assistance making life decision (treatment decision, potential support they may require during the treatment phase) Emotional coping Financial support (loss of work hours) Sexual intercourse Perceived loss of intimacy Support Spiritual needs (coping)	Clinicians (urologist) Healthcare professionnals Family (Partners) Work Church community	Denial, avoidance of the situation Urinary symptoms affected ability to socialise with family and friends Perceived loss of intimacy Difficulty to initiate the conversation about sexuality Isolation They would have liked a discussion and explanation of the findings immediately after their procedure, which did not always happen Lack of information Delay in waiting for their procedures	Active coping, acceptance of their condition and using sense of humour Sharing their sexual concerns with their partners was beneficial Family and support Subsequent follow-up consultation They reported that they would like to be involved in making their treatment decisions regarding when they will have their follow-up cystoscopy; other participants were happy to leave this to the urologist Call with the treating physician	High
Simard et al. (2013) [38]	USA (70 studies) UK (12 studies) Germany (10 studies) Australia (7 studies) Canada (7 studies) International (6 studies) Netherlands (5 studies) South Korea (2 studies) Iran (2 studies) Italy (1 study) New Zealand (1 study) Denmark (1 study) Norway (1 study) Taïwan (1 study) China (1 study) France (1 study) Japan (1 study) Thaïland (1 study)	130 included articles *N* = 46,487 participants Cross-sectional (*n* = 89) Longitudinal (*n* = 30) RCT (*n* = 7) Pilot study (*n* = 2) Mixed design (*n* = 2)	Marital status (Married of living with a partner) Had children Income Employment Ethnicity (non-white) Rural Appalachian Sexual problems Complementary alternative medecine use Healthcare consultation and health behaviour Healthcare satisfaction Distress Social support Self-efficacy Family functionning Meaning of illness Religiosity/spirituality Role functioning Social functioning Cognitive functioning Unmet Needs	Family Work Caregiver Alternative medicine Spiritual/Religious	Marital status (Married of living with a partner) Had Children Income Employment Ethnicity Sexual problems Complementary alternative medecine use Healthcare consultation and health behaviour Distress Unmet needs	Marital status (Married of living with a partner) Employment Healthcare satisfaction Social support Self-efficacy Social functioning Family functioning Cognitive functioning Religiosity/spirituality Beliefs about consequences of disease Reflection/Relaxation Coping and interpersonal styles of coping Positive meaning to the illness	High
Vivar et al. (2009) [39]	USA (35 studies) UK (8 studies) Canada (2 studies) Thaïland (2 studies) New-zealand (1 study) Japan (1 study) Singapore (1 study) Spain (1 study) Netherlands (1 study)	52 included articles *N* = 5672 patients Quantitative studies (*n* = 35) Review (*n* = 9) Qualitative review (*n* = 8)	Minor physical symptoms Medical follow-up Long term survivors Social support	Family Caregivers Partners	Dealing with the diagnosis of recurrent cancer Living with uncertainty Facing treatment again	Medical follow-up Social support Quality with partners	Moderate
Webb et al. (2023) [40]	USA (20 studies) Germany (11 studies) Taiwan (3 studies) Ireland (3 studies) China (2 studies) Canada (2 studies) UK (1 study) Netherlands (1 study) Turkey (1 study) Australia (1 study)	45 included articles *N* = 6172 caregivers Cross-sectionnal (*n* = 37) Longitudinal (*n* = 5) Psychometric scale evaluation (*n* = 2) RCT (*n* = 1)	Caregivers Dyadic relation between caregivers and survivors Protective role of caregivers	Caregivers	Dyadic relationship Avoidance of talking about distressing issues, like FCR	Understanding and treating FCR among caregivers	High
Williams et al. (2021) [41]	Australie (3 studies) USA (2 studies) Canada (2 studies) Germany (2 studies) Iran (1 study) Netherlands (1 study)	11 included articles *N* = 3151 participants Cross-sectionnal (*n* = 5) RCT (*n* = 4) Longitudinal (*n* = 2)	Higher healthcare satisfaction Higher healthcare usage Primary care appointments when high FCR FCR predicts visits the number of visits made to psychologists, dietitians and other allied care providers Maladaptive coping	Alternative medecine Spiritual/Religious	Institutional healthcare costs Inadequate institutional resources Maladaptive coping	CBT Care of patients	High
Yang et al. (2019) [2]	USA (9 studies) Germany (2 studies) Canada (2 studies) Netherlands (2 studies) Sweden (1 study) Finland (1 study)	17 included articles *N* = 4192 patients Cross-sectional (*n* = 14) Cross-sectional and follow-up studies (*n* = 3)	Being employed Struggle spirituality Psychological distress Social functioning	Work Spirituality	Being employed Type of cancer Struggle spirituality Psychological distress Social functioning	Counselors Religious peers Support groups Clinical psychologists AYA oncology/survivorship	High

Note: CBT: Cognitive Behavioral Therapy; NR: Not Reported; RCT: Randomized Control Trial.

**Table 2:** Quality appraisal results.

	Item Number of Checklist											
	1	2	3	4	5	6	7	8	9	10	11	Score
Anderson et al. (2021) [21]	Y	Y	Y	Y	Y	U	Y	Y	U	U	Y	8
Bamidele et al. (2022) [22]	Y	Y	Y	Y	Y	Y	Y	Y	NA	Y	U	9
Crist and Grunfeld (2013) [23]	Y	Y	Y	Y	Y	Y	U	Y	N	N	N	7
Dawson et al. (2016) [24]	Y	U	Y	Y	N	N	N	U	NA	Y	Y	5
Deckx et al. (2021) [14]	Y	Y	Y	Y	Y	Y	Y	Y	N	Y	Y	10
Gormley et al. (2022) [25]	Y	Y	Y	Y	Y	Y	Y	Y	N	Y	Y	10
Hampton et al. (2024) [26]	Y	Y	Y	Y	Y	Y	Y	Y	N	Y	U	9
Koch et al. (2013) [3]	Y	Y	Y	Y	N	N	Y	Y	N	Y	U	7
Lavery and O’Hea (2010) [27]	N	N	U	Y	N	U	N	N	N	U	Y	2
Lisy et al. (2019) [28]	Y	Y	Y	Y	Y	U	U	Y	N	N	Y	7
Liu et al. (2019) [29]	Y	Y	Y	Y	Y	Y	U	Y	N	N	Y	8
Lu et al. (2023) [30]	Y	Y	Y	Y	Y	Y	Y	Y	Y	U	Y	10
Luo et al. (2024) [31]	Y	Y	Y	Y	Y	Y	Y	Y	N	Y	N	9
McGeechan et al. (2022) [32]	Y	Y	Y	Y	U	Y	Y	Y	NA	N	Y	8
Mistchke (2008) [33]	U	Y	U	Y	N	N	N	U	N	N	Y	3
Neves et al. (2023) [34]	Y	Y	Y	Y	Y	N	Y	Y	N	N	Y	8
O’Rourke et al. (2021) [35]	Y	Y	Y	Y	Y	Y	Y	Y	N	Y	Y	10
Schmid-Büchi et al. (2008) [36]	Y	Y	U	Y	Y	N	N	N	N	Y	Y	6
Schubach et al. (2024) [37]	Y	Y	Y	Y	Y	U	Y	Y	N	Y	Y	9
Simard et al. (2013) [38]	Y	Y	Y	Y	Y	Y	Y	Y	N	U	Y	9
Vivar et al. (2009) [39]	Y	Y	N	Y	Y	N	N	Y	N	Y	Y	7
Webb et al. (2023) [40]	Y	Y	Y	Y	Y	Y	Y	Y	Y	Y	Y	11
Williams et al. (2021) [41]	Y	Y	Y	Y	Y	Y	U	Y	N	Y	Y	9
Yang et al. (2019) [2]	Y	Y	Y	Y	Y	Y	Y	Y	N	Y	U	9

Note: Four levels of assessment quality scores: Met (Y); Not Met (N); Unclear (U); and Not Applicable (NA). JBI critical appraisal checklist for systematic reviews and research syntheses: Q1: Is the review question clearly and explicitly stated? Q2: Were the inclusion criteria appropriate for the review question? Q3: Was the search strategy appropriate? Q4: Were the sources and resources used to search for studies adequate? Q5: Were the criteria for appraising studies appropriate? Q6: Was critical appraisal conducted by two or more reviewers independently? Q7: Were there methods to minimize errors in data extraction? Q8: Were the methods used to combine studies appropriate? Q9: Was the likelihood of publication bias assessed? Q10: Were recommendations for policy and/or practice supported by the reported data? Q11: Were the specific directives for new research appropriate?

**Table 3:** Search strategy by database.

Database	Records	After Duplicates	Removed by Titles and Abstracts	Reports Sought for Retrieval	Removed by Eligibility Criteria	Studies Included in the Review
PubMED	366	29 removed 337 left	265 removed 72 left	3 not retrieved 69 left	(i) : 5 (ii) : 4 (iii) : 9 (iv) : 36 15 left	*N* = 15
CINAHL	2192	175 removed 2017 left	1995 removed 22 left	1 not retrieved 21 left	(i) : 2 (ii) : 3 (iii) : 3 (iv) : 11 2 left	*N* = 2
PsycINFO	87	5 removed 82 left	41 removed 41 left	1 not retrieved 40 left	(i) : 4 (ii) : 5 (iii) : 4 (iv) : 23 4 left	*N* = 4
SocINDEX	337	7 removed 330 left	280 removed 50 left		(i) : 5 (ii) : 18 (iii) : 12 (iv) : 13 2 left	*N* = 2
PsycArticles	278	25 removed 253 left	162 removed 91 left		(i) : 70 (ii) : 1 (iii) : 3 (iv) : 17 0 left	*N* = 0
Business Source Premier	399	16 removed 383 left	343 removed 40 left		(i) : 36 (ii) : 0 (iii) : 0 (iv) : 4 0 left	*N* = 0
Grey literature and Citation searching	3	0 removed 3 left	0 removed 3 left		(i) : 0 (ii) : 0 (iii) : 0 (iv) : 2 1 left	*N* = 1
Total	*N* = 3659	*N* = 3402	*N* = 316	*N* = 311	*N*= 24	*N* = 24

Note. (i) Studies published in a language other than French or English or not focusing on cancer. (ii) Qualitative and quantitative studies. (iii) Letters to the editor, case studies, validation studies, RCT, or only meta-analysis. (iv) Not focusing on ecology and fear of recurrence of cancer.

## Data Availability

No new data were created or analyzed in this study.
